# The Enclosed Intestinal Microbiome: Semiochemical Signals from the Precambrian and Their Disruption by Heavy Metal Pollution †

**DOI:** 10.3390/life12020287

**Published:** 2022-02-15

**Authors:** David Smith, Miryam Palacios-Pérez, Sohan Jheeta

**Affiliations:** 1 Network of Researchers on the Chemical Evolution of Life (NoRCEL), Leeds LS7 3RB, UK; 2 Theoretical Biology Group, Institute of Biomedical Research, National Autonomous University of Mexico, Mexico City 04510, Mexico

**Keywords:** cancer, dysbiosis, epigenetics, exposome, gut–brain axis, ingestible sensor, maternal microbial inheritance, microbial sentinel cells, semiochemical, viral shunt

## Abstract

It is increasingly likely that many non-communicable diseases of humans and associated animals are due to the degradation of their intestinal microbiomes, a situation often referred to as *dysbiosis*. An analysis of the resultant diseases offers an opportunity to probe the function of these microbial partners of multicellular animals. In our view, it now seems likely that vertebrate animals and their microbiomes have coevolved throughout the Ediacaran–Cambrian transition and beyond, operating by semiochemical messaging between the multicellular host and its microbial community guest. A consideration of the overall role of the mutualistic intestinal microbiome as an enclosed bioreactor throws up a variety of challenging concepts. In particular: the significance of the microbiome with respect to the immune system suggests that microeukaryotes could act as microbial sentinel cells; the ubiquity of bacteriophage viruses implies the rapid turnover of microbial composition by a viral-shunt mechanism; and high microbial diversity is needed to ensure that horizontal gene transfer allows valuable genetic functions to be expressed. We have previously postulated that microbes of sufficient diversity must be transferred from mother to infant by seemingly accidental contamination during the process of natural birth. We termed this *maternal microbial inheritance* and suggested that it operates alongside parental genetic inheritance to modify gene expression. In this way, the adjustment of the neonate immune system by the microbiome may represent one of the ways in which the genome of a vertebrate animal interacts with its microbial environment. The absence of such critical functions in the neonate may help to explain the observation of persistent immune-system problems in affected adults. Equally, granted that the survival of the guest microbiome depends on the viability of its host, one function of microbiome-generated semiochemicals could be to facilitate the movement of food through the digestive tract, effectively partitioning nutrition between host and guest. In the event of famine, downregulation of microbial growth and therefore of semiochemical production would allow all available food to be consumed by the host. Although it is often thought that non-communicable diseases, such as type 2 diabetes, are caused by consumption of food containing insufficient dietary fibre, our hypothesis suggests that poor-quality food is not the prime cause but that the tendency for disease follows the degradation of the intestinal microbiome, when fat build-up occurs because the relevant semiochemicals can no longer be produced. It is the purpose of this paper to highlight the possibility that the origins of the microbiome lie in the Precambrian and that the disconnection of body and microbiome gives rise to non-communicable disease through the loss of semiochemical signalling. We further surmise that this disconnect has been largely brought about by heavy metal poisoning, potentially illuminating a facet of the exposome, the sum total of environmental insults that influence the expression of the genetic inheritance of an animal.

## 1. Introduction: Microbiome-Function Deficiency Disease

Although it is traditional to consider an “us versus them” approach to microbes, shifting the emphasis to symbiosis permits a new understanding of the relationships between gene-based lifeforms. Accordingly, the purpose of this article is to consider vertebrate evolution from the perspective of the microbiome in order to provide a theoretical underpinning for the understanding and, indeed, eventual treatment of non-communicable disease. In this regard, we have previously suggested that such disease arises by a breakdown of semiochemical-related host–guest communication in two specific ways: (a) a failure to initially establish a gut–brain axis leads to poor mental health, cardiovascular and circulatory disease, in addition to increased adiposity; and (b) a failure to engage with the developing neonate immune system leads to a range of atopic, autoimmune, and similar diseases, including a greater chance of developing cancer [1].

A key feature of this scheme is *maternal microbial inheritance*, in which microbes—not just bacteria—are transferred from mother to neonate by contamination during the process of natural birth. In some way as yet unclear, in this scheme, these microbes effectively calibrate the immune system of the neonate, giving it a head start to be able to tolerate the local microbial environment of the mother [1]. Clearly, the validity of this model would be enhanced if an evolutionary pathway leading to this type of symbiosis were to be shown to be available. In this respect, logic suggests that the microbiome first arose from the original microbial community that gave rise to multicellular creatures during the Precambrian. Note that essentially the same “accidental transfer” argument applies to the invertebrates, as well as the vertebrates, both ovi- and viviparous. It is interesting to note that, by contrast, if the seemingly accidental transfer of microbes from mother to child is interrupted by C-section, then the resultant babies only pick up their “expected” maternal microbiota after some weeks, often experiencing healthcare-associated pathogen colonisation in the interim [2].

**Hypothesis** **1** **(H1).** *Rather than being a collection of random bacteria, the microbiome can be considered to be related to the original microbial communities from which multicellular life forms first arose*.

Of course, the process of extrapolating back to the Precambrian from a hypothesis that is not fully accepted in the first place is fraught with difficulty. Nevertheless, following this hypothesis to its logical conclusion may eventually lead to fresh insights. In particular, it seems that the present emphasis upon the prokaryote, rather than the eukaryote, composition of the microbiome may be misplaced. The same line of reasoning is yielding fresh insights when applied to the genetic mechanisms behind the development and propagation of cancer [3].

Perhaps the simplest way to describe the enclosed intestinal microbiome of a vertebrate animal is as a partner occupying an ecological niche within the animal. However, even in the macroscopic world, the nature of the interaction between a species and its environment is a matter of debate [4]. Although we have referred to the microbiome as a cofactor for evolution [1], fundamentally, the question is of survival on a case-by-case basis, bearing in mind that the term “species” is a philosophical construct that is capable of various definitions and does not correspond to any specific physical reality [5].

As noted in a recent review, over the last 200 years, increasing amounts of medical intervention have been dedicated to countering the threat of non-communicable disease [6]. It seems that much of this effort is aimed at offsetting the effects of a steadily degrading microbiome, some of which could be due to heavy metal toxins trapped inside the intestine (see Section 9) [1].

## 2. Of Germs and Genes: The Invertebrates

Evolution is a process by which populations change over time due to the inheritance of beneficial variations; however, there has been debate about the role of symbiotic microbes as part of this process. Building on her success with the origin of the mitochondrion [7], Professor Lynn Margulis developed the term *holobiont* to represent both the host and its mutualistic microbes acting as a single evolutionary unit [8]. Although a full review of the term is outside the scope of this current work, in essence it refers to the observed relationship between microbes and multicellular species and the assumption that this relationship is important for the evolution of the symbiont [9,10]. As discussion continued, the argument moved from symbiotic species to their genes—the *hologenome* [11]—although not without debate as to the exact meaning of the terms employed [12].

Much of these discussions involved the invertebrates but without coming to any definitive conclusion as to how much speciation is provoked by microbial involvement. For example, studies on related members of the parasitic wasp genus *Nasonia* produced non-viable hybrids, seemingly because the parents carried different strains of the bacterial genus *Wolbachia*, whereas the hybrids themselves ended up with mixed microbes [13]. Interestingly, a counter argument was later presented, making the ostensibly simpler suggestion that the act of hybridisation somehow disables the immune system of the offspring so that *any* infection will kill them [14]. The position taken within this article, though unproven, is illustrated by point (b) above and suggests that the microbial expression of mobile genetic elements actually helps to establish the immune system of the animal. Accordingly, it is the lack of such assistance that leads to the death of the hybrids. In human terms, the hybrids suffer from a form of *dysbiosis*, which, while inconvenient for us, is fatal for the animal [15]. A recent observation confirmed that microbes may have a greater role than just that of the contaminant that they seem to be. While the incorporation of various *Wolbachia* genes into *Drosophila* is commonly encountered, one study on *Drosophila ananassae* reported that its genome contained the *entire genome* of the bacterium *Wolbachia*, presumably by microbe–animal horizontal gene transfer, though for what reason remains entirely unclear [16].

The nematode *Caenorhabiditis elegans* is the simplest animal available to modern researchers and the interactions between it and the bacteria, fungi, and viruses that inhabit its intestine are in the process of being investigated [17]. Interestingly, experiments where nematode strains were raised within different soil communities showed that these animals were selective in their uptake of environmental microbes and always included core species distinct to their strain. These microbes were shown to protect their own specific host from infection by pathogens but not those of different strains [18]. Of course, the value of *C. elegans* is its simplicity; it has been used as a model for study of the innate immune system [19] and for aspects of genetic research [20]. Equally, microbiome strain-specificity could also extend to humanity, for example, the observed relationship with blood group antigens [21].

## 3. The Vertebrates

Although it is not the purpose of this publication to draw attention to human non-communicable disease, the microbiome-function deficiency hypothesis briefly described in the Introduction explains the outcomes described above: malfunctioning of the gut–brain axis, leading to weight gain and poor mental health on the one hand and a poorly discriminating immune system on the other [1]. Perhaps of greater significance is the timing of the onset of human non-communicable disease. Epidemiological observations led David J. Barker to his view that such disease is primed by events early in life, writing a paper in 1990: “The fetal and infant origins of adult disease”, including both heart disease and schizophrenia [22]. He later concluded that the foetal aspects were likely to be more important, titling a subsequent paper, “Fetal origins of coronary heart disease” [23]. Later researchers likewise focused on the in-utero aspects, stressing the correctness of the epidemiological arguments but without agreeing on a valid rationale for Barker’s observations [24,25]. By contrast, our microbiome-function deficiency hypothesis essentially relies on disease being primed from the very beginning of independent life, if not in the foetus, certainly from the neonate and possibly extending into early adulthood as the brain finally matures [1]. Immune conditions also start early, developing through a characteristic series described as the atopic march [26].

Possibly barring poor mental health, all these consequences are also found in domesticated and pet animals; however, as stated in an influential paper by Denis Burkitt, none has been described in the wild [27]. It is possible that lead poisoning, whether overt or covert via the microbiome, may be a part of the explanation for the sudden disappearance of hedgehogs, specifically *Erinaceus europaeus*, in the more heavily polluted parts of England [15]. More credibly, perhaps, the use of antibiotics to drive growth in farm animals prior to slaughter, not only in mammals such as swine [28] but also chickens [29] and possibly fish [30], may be indicative of such microbial malfunction [1]. Evidence of microbiome-function deficiency disease in domesticated animals is not restricted to weight gain, as atopic disease is also observed [31].

Studies on the bacterial microbiome of the cowbird, *Molothrus ater*, showed considerable variation between individuals, and it has been reported that the range of microbial diversity within these brood-parasitic birds and their passerine hosts can be greater than across all the mammal species put together [32]. Interestingly, these authors tested both a “nature hypothesis”, where the genome of the bird directs the microbiota, and a “nurture hypothesis”, where the host is more important. They found neither to be true but rather that location, diet, and age were the most significant features [32]. Although at first sight this is inconsistent with the idea of a single thread passing from the Precambrian to present times, the dilemma may be resolved if the focus is on microbial *function* rather than microbial *composition*, especially bearing in mind the possibility that it is the retention of microeukaryotes that is important [28,29,30,31,32,33].

A similar situation may pertain to people. *Bifidobacteria* have been suggested as potentially marking a healthy microbiome, though the evidence is not clear [34]. Conversely, the Hadza, considered to be a healthy population, were reported not to contain any *Bifidobacteria* within their microbiome [35]. It has already been remarked upon that the focus on prokaryotes, especially the economically important “probiotic” bacteria, downplays the potential significance of the unicellular eukaryotes that are known to be present in animal microbiomes [36].

## 4. Flexibility and Diversity: Germs in the Environment

The single most consistent finding relating to the absence of non-communicable disease is high microbial diversity [37]. Stool consistency, bacterial growth rates, and high gut motility are also associated with high microbial “richness” [38], which we describe as increasing the efficiency of energy flow through body and microbiome [1].

**Figure 1:**
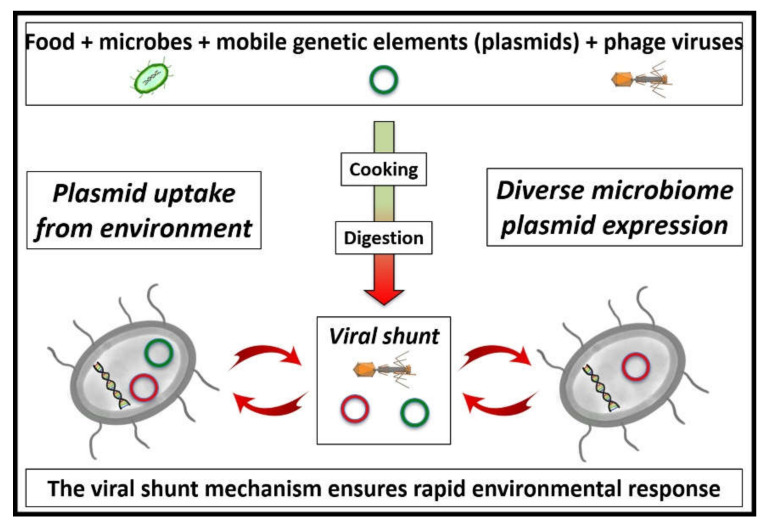
The viral-shunt mechanism within a diverse microbiome. In addition to nutrition, food brings in external microbes and their mobile genetic elements, along with phage virions. Both external and pre-existing phage particles act to disassemble microbes, leaving debris suitable for recycling into new entities [39,40]. With adequate diversity [37,38], this allows a rapid turnover of species to those more appropriate for changing circumstances and allows for redistribution of plasmids and other mobile genetic elements [1].

In the environment of enclosed microbial colonies, phage attack leaves debris in a form that can quickly be reassembled into new entities [39]. This process was first observed in the sea, trapping carbon in the upper layers of the ocean, and was described as a *viral shunt* [40]. Uptake of mobile genetic elements by transformation has been described as being facilitated by “superspreader” bacteriophages [41]. In this way, microbiota update their toolkit, incorporating novel abilities, such as antibiotic resistance, for example. Presumably, the ability to digest seaweed glycans amongst Japanese people came about by the same “extrinsic enzyme-updating” mechanism [42]. As stated above, the greater the diversity found within the microbiome, the healthier the population, as exemplified by the Hadza [35]. Although surprising at first sight, given the tendency for evolutionary processes to pare away anything not strictly necessary for survival, it is possible that this diversity enables the flexibility necessary for continued operation in changing circumstances (Figure 1).

Bacterial diversity also seems to be important within the context of pancreatic tumours, both in the intestinal microbiome of the sufferer and *within* the tumour itself [43]. Apparently, the rare examples of slower tumour growth are not associated with the genetic makeup of the tumour but rather with increased microbial diversity. Significantly, mice bearing human pancreatic tumours were protected from the rapid growth of these tumours if the intestinal microbiome of these rare long-term survivors of the human disease were transferred to them by faecal microbiota transplantation [43]. Regrettably, no evidence was gathered concerning the presence of unicellular eukaryotes within either the microbiome or the tumour itself.

## 5. Parallel Interactions within the Intestinal Host-Guest Relationship

Instead of treating the role of the microbiome simply as the sum of its individual components, we considered the properly functioning system as if it were a single mutualistic community with a defined primary function: to direct the immune system of the host from the time of birth [33]. In this context, we use the word *semiochemical* to represent chemicals conveying a message from one organism to another, bearing in mind that mutualism requires parallel quid pro quo interactions.

**Figure 2:**
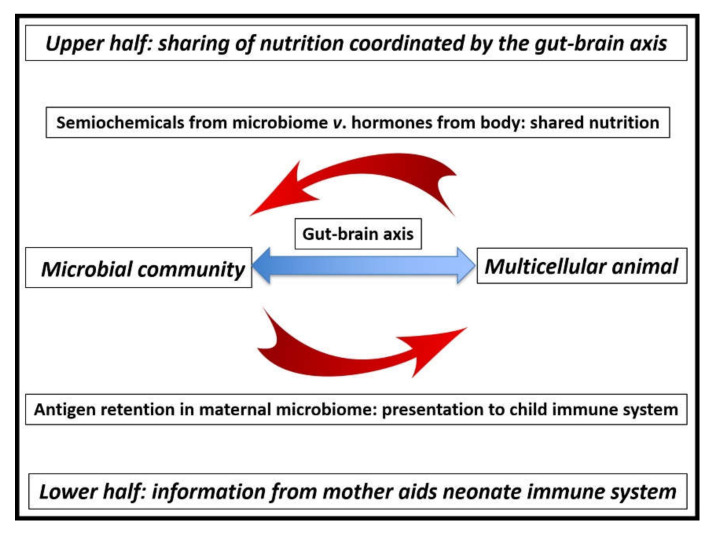
Summary of mutualistic interactions between host and guest. The ability to share nutrition mediated by the gut–brain axis (top half of the diagram) is complemented by the value of the microbiome in calibrating the immune system of the neonate to tolerate the microbial environment of the mother (bottom half of diagram). A failure of the microbiome gives rise to the characteristic mix of non-communicable diseases: weight gain and poor mental health from malfunctioning semiochemical production, as well as immune-system problems [15].

Figure 2 illustrates the ideal host/guest relationship, in which nutrition is partitioned on the one hand (top half) and the microbiome supplies the neonate with immune-system information on the other (bottom half). When fully functioning, the relationship is seamless, and the microbiome is effectively invisible [33].

### 5.1. Semiochemical Production

Such is the importance of small-molecule synthesis within the microbiome that it has been classed as an endocrine organ in its own right [44]. Its output falls into two broad classes: those associated with energy metabolism [45] and those associated with interkingdom signalling, such as the catecholamines [46]. The former includes both vitamins and short-chain fatty acids (SCFAs), alkanoic acid salts bearing from two to five carbon atoms. Appreciably water-soluble, as they diffuse out of the intestinal lumen, they make a valuable contribution to the energy requirements of nearby cells, as well as having broader systemic effects [47]. Interestingly, they also affect the immune system, at least within the colon [48]. Although they may not be semiochemicals themselves, they are reported to activate serotonin-producing cells [49], thus having an indirect signalling effect. By contrast to the SCFAs, substances such as dopamine and serotonin, although first recognised as neurotransmitters, are now known to have hormone-like signalling properties, having a role both in the brain [50] and the periphery, the latter reportedly controlled by gut microbes [51]. When produced inside the gut lumen, these molecules may be examples of semiochemical signalling molecules [46], not working by penetrating the brain directly but by sending messages via the gut–brain axis [33].

### 5.2. The Microbiota (or Microbiome)–Gut–Brain Axis

While the nervous system connects all parts of the body to the brain, it is only recently that the unusual nature of the range of gut–brain “axial” connections has been realised. The key experiments involved the raising of so-called germ-free mice and the realisation that their brain chemistry, and hence behaviour, were significantly affected by the absence of microbes at the moment of birth, an effect partly overcome by their subsequent supply [52]. A similar unusual connectivity was found to exist between the brain and another germ-rich organ, the skin, although in this case the communication appeared to be three-way, also involving the gut and its microbes [53]. Of the various microbiomes associated with the human body, that in the enclosed intestine seems to be the master [54]. Although the exact details remain elusive, a problem with the microbiota–gut–brain axis is reported to be connected to the development of obesity [55]. Needless to say, disruption of the enclosed intestinal microbiome is also associated with immune-system problems, as illustrated by a recent article on multiple sclerosis, asking the question about the causative relationship between immune-system disease on the one hand and a malfunctioning gut–brain axis on the other [56]. The microbiome-function deficiency hypothesis described in the present and previous articles [1] suggests that the two exist in parallel, albeit initiated by microbiome dysfunction during early childhood. This latter effect would, if confirmed, be the infant equivalent of Barker’s “fetal origins hypothesis” [22].

### 5.3. Regulation of the Flow of Nutrition

Movement of food from the upper gut to the microbiome depends on the exact balance of chemical signals: semiochemicals from the microbiome and hormones from the body itself (Figure 2). If all is well and there is adequate microbial diversity [37], food passes down to the microbiome, allowing microbial growth and its subsequent excretion [38]. If famine strikes, an increase in the levels of the appropriate hormones allows the body to retain nutrition by overwhelming the effect of microbial semiochemicals. In these circumstances, microbial growth is slowed, but the relevant entities remain viable so as to be reactivated when the emergency passes [1]. Conversely, pregnancy initiates a precise series of actions, allowing the microbiome to be passed on to the neonate during the process of natural birth [57]. As illustrated in Figure 1, mobile genetic elements and phage virions pass down to the microbiome alongside nutrition.

### 5.4. Maternal Microbial Inheritance

Growth processes require extensive but temporary control of gene expression at key stages, for which the term epigenetics has been coined [58]. More recently, paternal epigenetic inheritance has been invoked [59], but, interestingly, genetic factors do not seem to account for a substantial portion of the known inheritance of complex phenotypes, a phenomenon that has been described as “missing heritability” and which may partly involve heritable epigenetic modifications [60]. By contrast, the entire notion of human epigenetic inheritance has been questioned on the grounds of inadequate evidence [61]. Significantly, a recent comprehensive review of infant development stressed the need for efficient transfer of a high-diversity microbiome from the mother to the child to avoid the later onset of a wide range of diseases [62]. It is probable that communication between the microbiome and its host influences the growth process. In this context, microbial epigenetic involvement has been reviewed, albeit merely emphasising the fact that many gaps remain [63]. As is normally the case, sadly, all of the above quoted microbial studies referred only to bacteria, and the possible existence of mutualistic unicellular eukaryotes was not considered.

### 5.5. Microbial Sentinel Cells

As stated above, there is evidence that SCFAs can act as a source of energy for the colonic immune system [48], so, in principle, microbiome-function deficiency could partly disable the immune system, initiating the observed atopic and similar diseases of Mammalia [31]. As an alternative, however, it is possible that information is somehow held within the microbiome, ready to be passed on to the neonate, preparing it for the microbial environment in which its mother flourished [33]. Although there is no current evidence of such behaviour, nevertheless, this would be the most direct way to transfer antigens from mother to child, labelled either as “neutral” or as “potential foe”. In this way, the sudden exposure of an adult to a completely different suite of environmental microbes of unknown pathogenicity may generate the defensive, better-safe-than-sorry response of traveller’s diarrhoea [64]. As such a function would be likely to exceed the capability of prokaryote cells, it is possible that it may reside within one of the microeukaryote entities known to be present in the microbiome [36]. Indeed, there is a number of different varieties of protozoa that are increasingly coming under scrutiny. They include the profligate genus *Blastocystis*, a persistent and diverse range of microbial species, which, although they may be pathogens, can also be found in apparently healthy humans [65]. Their presence and activity can be shown to respond positively to plant spices and negatively to antibiotics [66]. Significantly, perhaps, they exist in many other creatures throughout the animal kingdom and can swap between different hosts with relative ease [67].

## 6. Semiochemicals: From Single Cells to Multicellular Animals

It is reported that the enzymatic mechanisms for the production of messenger chemicals could have been transferred to animals from bacteria via horizontal gene transfer, not only dopamine and serotonin, for example, but also histamine, acetylcholine, and nitric oxide [68]. Interestingly, some of these transfers apparently took place after the divergence of animals from fungi but all, seemingly, after the acquisition of the enzymes associated with the central metabolic pathway [68]. Although not definitive, the implication is increasing levels of microbe–animal communication, with these molecules being potential semiochemical messengers for communication between the enclosed intestinal microbiome and the vertebrate animal. Potentially defined as a major evolutionary transition [69], it is possible that this development allowed early animals to transition across the Ediacaran–Cambrian boundary [70].

The presence of semiochemicals, such as dopamine, in the mammalian gut lumen suggests the possibility of assessing microbiome effectiveness. In principle, an ingestible sensor, a pill-like device bearing a detector and a transmitter, could be developed so as to measure the levels of such species upon exposure to foodstuffs containing substances such as dietary fibre and polyphenols, for example. The resultant response may indicate the degree of dysbiosis and, with help of “omic” approaches [71,72], generate personalised, accurate, and potentially massively applied treatments to assist in finding a method of amelioration, if not an actual cure [73].

## 7. Microbial Sentinel Cells: Potential Partners of the Immune System

Dendritic cells are antigen-presenting cells associated with both the induction of primary immune responses and with immunological tolerance [74]. It is possible that these and similar agents evolved from microeukaryotes present within early animals: microbial sentinel cells, as described above using the example of the *Blastocystis* genus [1].

The domains of Archaea and Bacteria are collectively prokaryotes [75,76,77], emerging from the last universal common ancestors (LUCAs), whereas the third domain, Eukarya, a chimera of Archaea and Bacteria, appeared later. Lynn Margulis (then Lynn Sagan) also backed the concept of three domains of life in her published paper entitled, “On the origin of mitosing cells”. She put forward the inspired notion that eukaryotes were the product of a symbiotic union of an archaeon and a bacterium [7,78,79,80], forming what Carl Woese later described as the third domain of life [81]. The early development of these unicellular entities was dominated by extensive horizontal gene transfer, natural selection only taking place above a certain level of complexity, described by Woese as the *Darwinian Threshold* [76,81,82,83]. Among the many unicellular eukaryotes currently existing, perhaps the most striking example is *Toxoplasma gondii*, with its influence upon the brain of rodents, causing them to exhibit inappropriate behaviour in the presence of cats [84]. It is likely that the first eukaryotic organisms played some sort of commanding or coordinating roles within the prokaryote communities in which they arose, thereby ensuring their own competitive advantage and allowing diversification into multicellular animals [85]. This process is still underway [86].

Subsequent evolutionary milestones include the appearance of bilaterians [87] and Cambrian vertebrates [88]. Since the discovery of a Precambrian fossil in the 1950s by school children in England [89], knowledge of the surprising body shapes of the “enigmatic” Ediacaran biota has increased considerably, including confirmation of the presence of animal-like entities by detection of steroids [90]. It is possible that the viral-shunt mechanism (Figure 1) and mutualistic microbiome (Figure 2) were already in operation during these times [68]. Of course, the virus/cell relationship predates eukaryotes and has operated since the dawn of life [91,92,93].

## 8. The Loss of Immune-System Selectivity

In his alliteratively entitled paper, “Hay fever, hygiene and household size”, David Strachan drew attention to the apparently inexplicable rise of immune-system disturbances, such as seasonal allergic rhinitis (hay fever). He suggested that the overuse of antimicrobial cleaning agents reducing our exposure to environmental microbes might be the cause [94] but it was later concluded that this was unlikely to be the whole truth [95]. At the time, however, the concept excited significant interest, so that when Rook and his team attempted to identify specific external entities playing a role in this training process, they used the term “old friends” [96]. It may be that such an *intrinsic* microbial sentinel-cell function explains their difficulties, as they were looking for an elusive *extrinsic* microbial factor [96]. It is possible to envisage a role for microeukaryote sentinel cells to probe suspect particles within the gut lumen to see if they are friend or foe, a normally harmless food protein or a harmful bacterium, for example, and somehow pass that information *from the microbiome of the mother* on to the immune system of the neonate. 

In the early 19th century, John Bostock made the first detailed description of the disorder that was later to become known as hay fever, a characteristic condition indicating a malfunctioning immune system, predating the development of antiseptics or antibiotics by at least 40 years [97]. In Bostock’s day, such *catarrhus aestivus* was very rare and, as he makes clear, was restricted to the upper classes [98]. By its very nature, the mammalian intestine is effectively a closed bioreactor with infrequent emptying. Noting that the cosmetic preparations of those days could contain heavy metal salts [99], we have suggested the possibility of the poisoning of the microbiome by sparingly soluble toxic metal particles that are not readily absorbed into the body [15]. In Bostock’s case, such poisoning would have taken place within his mother, leaving him with the propensity for non-communicable disease after microbial inheritance of her own poorly performing microbiome. Industrialisation of society has left a residue of heavy metal pollution [100], while attention is also being drawn to its toxic effects on animals nearer the base of the food chain [101]. As noted above, the loss of microbe–animal cooperation in insects may lead to their death [13,14], and it could also contribute to the spread of non-communicable disease in our own species [6].

## 9. Environmental Insults and the Microbiome

In recent years there has been increasing interest in the concept of the exposome: exploring external challenges to health through the interaction of pollution with the human genome [102,103]. A recent article suggested guidelines to the consequences of such damage in the form of “hallmarks of environmental insult” [104]. Although the general expression “altered microbiome communities” is included among these hallmarks, the intermediacy of the microbiome between pollutant and genome potentially adds an extra layer of understanding to the consequences of environmental insult. 

Although sufficiently diverse intestinal microbiota are expected to be efficient at metabolising many substances, exposure to heavy metal ions could be exceptionally dangerous, especially within the context of viral-shunt-induced rapid microbial turnover, and such disruption is indeed observed [105]. Of course, many metal ions are likely to be toxic to microbes, and some are currently being considered for commercial antimicrobial use, including those used in catalytic converters, for example [106]. These metals are now widely distributed in the environment [107]. Although lead has largely been removed from petrol [108], the danger of heavy metal pollution is far from over. In particular, antigen-probing eukaryote microbial sentinel cells, if they exist, may be expected to be particularly vulnerable to the effects of heavy metal poisoning within the enclosed intestinal environment. The disappearance of these still-hypothetical entities may represent the most succinct way to explain the prevalence of immune-system disease in heavily pollution-exposed populations following industrialisation. Under these circumstances, maternal microbial inheritance may well lead to a situation in which damage to the microbiome is not readily reversed.

## 10. Cancer

The recent rise of cancer is a major concern, albeit with manifold apparent causes [109]. One explanation for this increase is the inability of a compromised immune system to eliminate precancerous cells, i.e., that the friend/foe detection system is no longer as effective as it used to be [15]. However, the tumour microbiome study quoted above [43] offers a complementary explanation: that the reduced microbial diversity within the intestine compared to, for example, that of the Hadza people [35], implies the loss of the ability to moderate the growth of tumours. Of course, it is possible that both factors are at work, producing a greater frequency of tumours with a greater growth rate [109].

In the light of the recent observations on pancreatic cancer [43] and the arguments presented above, it may be that we should be considering an extended oncogene suite in which the presence of suitable microbes effectively provides a surrogate microbiome, hopefully toning down the growth of the cancer. It is possible that, by analogy with *C. elegans* noted above [18], microbes can form mutualistic associations with specific tumours, *almost as if the tumours themselves were behaving as incipient animals*. In accordance with the above interpretation, in his magisterial review of the literature, Sinkovics [3] commented on the propensity of cancer cells to behave as if they were devolved amoeba-like entities, expressing survival systems that are derived from ancestral unicellular protists [110].

In this article, our argument has been that the poorly studied unicellular eukaryotes have a much greater role than has been assigned to them so far [31,32,33,34,35,36], as exemplified by the spectacular ability of *T. gondii* to control rodent behaviour to its own advantage [84]. In this context, Woese’s observation of the role of horizontal gene transfer within unicellular eukaryotes operating below the Darwinian Threshold may well prove to be especially relevant to their role within the microbiome [76,81]. Bearing in mind the extent of heavy metal pollution, the original behaviour of the human microbiome may only be found in relatively unexposed populations. Such people already in cooperation with medical science include the Hadza [35] or, more recently, the Tsimane of the Bolivian Amazon [111]. The search for a method of compensation for valuable information gained from indigenous people has led to the “principle of reciprocity”, and this could be usefully employed here [112].

## 11. Conclusions: The Amelioration of Non-Communicable Disease

Although the basic scheme concerning the dual action of the microbiome upon the neonate seems to be consistent with the bulk of observations, more work is needed to confirm the existence of microbial sentinel cells in populations unaffected by non-communicable disease. Specific actions to ameliorate such disease could include the following:

(a) Develop methods to check the microbiome function of people not exposed to heavily polluted environments and;
(b) Confirm the role, if any, of unicellular eukaryotes, as well as prokaryotes. Finally,
(c) Consider the benefits of an ingestible sensor for the measurement of semiochemicals in order to assist the amelioration of any microbiome-function deficiency in individuals and their children.

Bearing in mind maternal microbial inheritance, however, once the microbiome has lost functionality, it is unlikely that the situation can be remedied without some kind of microbial supplementation during the birth process, possibly including unicellular eukaryotes. Although non-human experiments would have to be performed, this should not cause distress to the animal. Interestingly, it may well prove feasible to employ a generic microbial supplement applicable throughout Mammalia, maintaining maximum diversity and the potential for flexibility while allowing the body to determine its own preferred level of interaction.

Nevertheless, no matter how efficient the microbiome, note that it is still vulnerable to interruption at critical stages, for example, if it is temporarily disabled by antibiotic use. This may be especially significant during the development of the gut–brain axis and the growth of the brain. We hope to cover subjects such as the distribution of nutrition and, separately, poor mental health in subsequent articles.
